# Exogenous Polyamines and Plant Salinity Tolerance: A Systematic Review, Evidence Map, and Family-Aware Quantitative Synthesis

**DOI:** 10.3390/antiox15091165

**Published:** 2026-09-14

**Authors:** João Everthon da Silva Ribeiro, Toshik Iarley da Silva, Ester dos Santos Coêlho, Jucilene Jesus Santos, Adriel Sousa Matos Silva, Elaine Conceição Gomes, Pablo Henrique de Almeida Oliveira, Aftab Jamal, Aurélio Paes Barros Júnior

**Affiliations:** 1 Center for Agricultural, Environmental and Biological Sciences, Federal University of Recôncavo da Bahia, Cruz das Almas 44380-000, Brazil; toshik@ufrb.edu.br (T.I.d.S.); jucysantos@aluno.ufrb.edu.br (J.J.S.); adrielsousam@aluno.ufrb.edu.br (A.S.M.S.); lanegomes52@hotmail.com (E.C.G.); 2 Department of Agronomic and Forest Sciences, Federal Rural University of the Semi-Arid Region, Mossoró 59625-900, Brazil; estersantos12@hotmail.com (E.d.S.C.); pabloalmeidaagro@gmail.com (P.H.d.A.O.); aurelio.barros@ufersa.edu.br (A.P.B.J.); 3 Department of Soil and Environmental Sciences, Faculty of Crop Production Sciences, The University of Agriculture, Peshawar 25130, Pakistan; aftabses98@gmail.com

**Keywords:** putrescine, spermidine, spermine, oxidative stress, reactive oxygen species, ion homeostasis, family-aware quantitative synthesis

## Abstract

Salinity disrupts plant redox balance, ion homeostasis, and water relations, and exogenous polyamines have been widely investigated as potential mitigators of these effects. However, it remains unclear which polyamine-mediated responses are reproducible across independent studies and whether redox protection consistently translates into downstream physiological recovery. We therefore conducted an integrated systematic review, evidence map, and publication-family-aware quantitative synthesis to evaluate the effects of exogenous putrescine, spermidine, and spermine in salt-stressed plants. We systematically searched Scopus and Web of Science through 9 August 2026, and eligible studies compared exogenous-polyamine-treated plants with corresponding salt-stressed controls. The review was not prospectively registered. Of 1093 records, 647 unique records were screened, and 179 reports were assessed in full text, yielding 34 retained reports and 444 complete quantitative contrasts. Multilevel random-effects models estimated reductions of 19.2% in malondialdehyde (95% confidence interval [CI], 12.9–25.1%) and 25.6% in hydrogen peroxide (H_2_O_2_; 95% CI, 15.5–34.6%). Superoxide showed a protective mean response but remained uncertain because its 95% CI included the null. In contrast, antioxidant-enzyme responses varied widely across studies, with no consistent cross-family increase in superoxide dismutase (SOD), catalase (CAT), ascorbate peroxidase (APX), or peroxidase (POD/POX) activity. Conservative independent-family syntheses supported favorable ion balance, modestly higher relative water content, and greater dry biomass, whereas total chlorophyll remained uncertain and net photosynthesis was highly heterogeneous. No clear superiority of putrescine, spermidine, or spermine was detected. Overall, exogenous polyamines most consistently reduced oxidative damage and stabilized ion–water homeostasis, whereas antioxidant–enzyme activity and photosynthetic recovery were more context-dependent. Interpretation is limited by small independent-family counts, incomplete reporting in some studies, and the absence of a formal study-level risk-of-bias assessment. By integrating systematic review, evidence mapping, and family-aware quantitative synthesis, this review provides an evidence-based hierarchy that distinguishes reproducible polyamine-mediated responses from context-dependent outcomes and helps define priorities for future salinity-tolerance research.

## 1. Introduction

Soil and irrigation–water salinity impose a compound stress on plants rather than a single physiological lesion. The primary osmotic constraint rapidly restricts water acquisition and stomatal conductance, while prolonged exposure promotes Na^+^ and Cl^−^ accumulation, K^+^ displacement, nutrient imbalance, membrane destabilization, photochemical impairment, and excessive formation of reactive oxygen species (ROS). These processes reinforce one another: ionic imbalance can intensify oxidative pressure, oxidative damage can compromise membrane selectivity, and restrictions on carbon assimilation can alter both energy status and antioxidant demand. Consequently, a successful salinity-mitigation strategy must be evaluated across several connected levels of organization [1,2,3].

Polyamines are small aliphatic polycations with established roles in growth, membrane and macromolecule stabilization, ion transport, stress signaling, and redox regulation. Putrescine (Put), spermidine (Spd), and spermine (Spm) are the most frequently studied plant polyamines. Exogenous application through foliar spraying, seed priming, root-zone delivery, or nutrient solutions has been reported to modify antioxidant systems, reduce lipid peroxidation, influence ROS signaling, improve K^+^/Na^+^ balance, preserve tissue hydration, protect pigments and photosynthetic machinery, and sustain growth under salt stress. However, the literature is highly heterogeneous in species, dose, salinity level, exposure time, sampling organ, application method, and endpoint selection [4,5,6,7]. Endogenous polyamine homeostasis is dynamic, and externally supplied polyamines enter pre-existing metabolic and transport networks rather than acting independently. Consequently, their effects may depend on endogenous polyamine turnover, cellular redistribution and accumulation, and the physiological identity of the responding tissue [5,8,9,10].

However, changes in polyamine abundance cannot be interpreted as a simple biochemical index of tolerance. Long-term salinization of contrasting rice cultivars caused spermine accumulation in both genotypes, yet experimentally increasing spermine through inhibition of spermidine synthase did not reduce salt injury, indicating that accumulation alone is not sufficient to establish a causal tolerance trait [8]. At the membrane level, the effect of exogenous polyamines is similarly conditional. In maize and *Arabidopsis* roots, Put, Spd, and Spm reduced NaCl-induced K^+^ loss in mature root tissues but intensified K^+^ efflux in the distal elongation zone, demonstrating a narrow spatial and physiological window in which polyamine-mediated ion retention is beneficial [9]. These findings help explain why superficially similar exogenous treatments can produce beneficial, neutral, or even adverse responses across experimental systems. These context-dependent responses suggest that changes in polyamine abundance become physiologically meaningful through spatially organized transport, oxidation, and signaling processes, particularly at membrane–apoplast interfaces [9,10,11].

Importantly, polyamine-mediated redox regulation is not limited to direct ROS scavenging. Polyamine oxidation can generate hydrogen peroxide (H_2_O_2_) as a localized signal, creating a mechanistic link between polyamine metabolism and stress-induced redox signaling. In tobacco, stress-induced spermidine export to the apoplast and subsequent oxidation generated H_2_O_2_ signatures associated with acclimatory responses, while in tomato, SlPAO4-dependent H_2_O_2_ production was required for the full spermidine-induced antioxidant response under saline–alkaline stress. These observations support a biphasic interpretation in which an early, spatially controlled H_2_O_2_ signal can activate defense, whereas sustained ROS accumulation reflects oxidative burden and damage [10,11]. Accordingly, ROS abundance should not be interpreted as synonymous with cellular injury: the biological meaning of H_2_O_2_ depends on its timing, localization, and persistence, whereas damage-associated endpoints such as malondialdehyde (MDA) represent a different level of the stress response.

Polyamines also intersect with nitrosative and hormonal signaling. Spd and Spm can rapidly stimulate nitric oxide production in *Arabidopsis* tissues, providing a direct link between polyamine status and NO-dependent signaling [12]. Under salinity, citrus plants treated with polyamines showed extensive reprogramming of oxidative and nitrosative status together with changes in the stress-responsive proteome, suggesting that the response extends well beyond individual antioxidant enzymes [13]. More recently, a genetically resolved tomato pathway connected jasmonate signaling with Spd synthesis and ion transport: the WRKY transcription factor SlWRKY81 directly activated spermidine synthase 2 (SlSPDS2) and the Na^+^/H^+^ exchanger SlNHX4, increasing Spd accumulation, K^+^ uptake, and Na^+^ efflux under saline–alkali stress [14]. Thus, polyamines are better viewed as components of an interacting redox-ion-hormone network than as isolated antioxidant metabolites.

Comparing Put, Spd, and Spm is biologically relevant because these polyamines differ in molecular charge, endogenous metabolism, cellular distribution, and signaling behavior. However, their apparent effects under salinity are also strongly conditioned by dose, species or genotype, tissue, stress intensity, and exposure duration, making it difficult to separate compound-specific effects from experimental-context effects [8,9,15,16].

This heterogeneity creates two limitations in the existing literature. First, narrative conclusions can overemphasize individual positive experiments without quantifying how reproducible the response is across independent study families. Second, a simple pooled meta-analysis can obscure mechanistic structure because multiple doses, tissues, time points, and outcomes within the same experiment are biologically and statistically dependent. The problem is compounded by evidence that polyamine effects can vary by tissue, dose, and exposure duration: cation retention may improve in one root zone but worsen in another. In contrast, supra-optimal polyamine exposure can inhibit growth through ROS- and NO-related signaling [1,9,15,16]. The present study was therefore designed as an integrated systematic review rather than a meta-analysis alone. The systematic-review and evidence-map components identified and characterized the eligible evidence base, whereas the quantitative synthesis was restricted to outcomes and contrasts that could be harmonized numerically. Within the quantitative component, we used a family-aware strategy to account for dependence among related comparisons. In practical terms, multiple contrasts arising from the same experiment or closely related publication family were not treated as fully independent observations, thereby reducing the disproportionate influence of studies that reported many doses, tissues, time points, or treatment combinations.

The central knowledge gap is whether the reported benefits of exogenous polyamines are reproducible across independent studies and whether improvements in redox status consistently propagate to ion–water homeostasis, photosynthetic recovery, and growth. We hypothesized that exogenous polyamines would produce more consistent favorable effects on direct oxidative-damage markers and ion–water homeostasis than on antioxidant–enzyme activities, photosynthetic performance, and growth, which were expected to show greater between-family variability because they integrate more context-dependent processes. We further hypothesized that no single polyamine—Put, Spd, or Spm—would show consistent superiority across experimental contexts. Accordingly, the primary objectives were to (i) quantify the effects of exogenous polyamines on recurrent oxidative-damage markers under salinity; (ii) determine whether antioxidant–enzyme activities show consistent cross-study responses; (iii) synthesize evidence for ionic and water homeostasis, photosynthetic performance, and biomass; (iv) explore whether Put, Spd, and Spm show evidence of different average effects; and (v) identify the experimental conditions and knowledge gaps that determine whether redox and homeostatic benefits translate into functional recovery.

## 2. Materials and Methods

### 2.1. Review Design, Search Strategy, and Eligibility

We developed the review as an integrated systematic review, evidence map, and family-aware quantitative synthesis of experiments in which an exogenous polyamine was applied to plants exposed to salinity or salinity–alkalinity stress.

This systematic review was conducted and reported in accordance with the Preferred Reporting Items for Systematic Reviews and Meta-Analyses (PRISMA) 2020 statement [17]. Searches were performed in Scopus and Web of Science. The final search exports contained 638 Scopus records and 455 Web of Science records (1093 records in total). After deduplication, 647 unique records proceeded to title and abstract screening. The review was not prospectively registered, and a stand-alone protocol was not prepared; eligibility, effect-size, family-assignment, and quality-control decisions were instead documented in frozen audit files before the final synthesis.

Studies were eligible when Put, Spd, Spm, or a clearly defined related polyamine was applied exogenously and when a salt-stressed polyamine-treated group could be compared with a corresponding salt-stressed control. Studies in which polyamines were only measured as endogenous responses, rather than manipulated as the exposure, were excluded from the causal review. Combination treatments were included in the primary synthesis only when a polyamine-alone arm was isolatable. Studies combining salinity with an additional stressor were eligible when the polyamine effect could be isolated against a matched control exposed to the same stress combination. Multiple genotypes, doses, tissues, salinity levels, and time points were retained during extraction and were treated as dependent effects when they originated from the same experimental family. The eligibility criteria applied during study selection are summarized in Data S1, Table S11.

Eligibility for the systematic-review layer and readiness for quantitative synthesis were treated as separate decisions. A study could remain biologically eligible when it reported a relevant exogenous-polyamine contrast but lacked recoverable dispersion, used an endpoint that could not be harmonized with the primary quantitative outcome, or contained a factorial structure that required conditional interpretation. Conversely, records were excluded from the causal synthesis when the experimental intervention was not a polyamine treatment even if endogenous polyamines were measured as physiological responses. This distinction prevented quantitative reporting quality from redefining the biological review question. Accordingly, the evidence was organized into linked but non-equivalent levels. The systematic-review corpus comprised all reports meeting the biological and causal eligibility criteria. The evidence map characterized the outcome domains represented across this corpus regardless of whether individual endpoints were quantitatively synthesizable. The quantitative synthesis was restricted to contrasts for which numerical outcomes could be harmonized and the required statistical information could be recovered with sufficient confidence. Finally, the integrated synthesis interpreted these quantitative estimates together with the broader eligible physiological and mechanistic evidence. Inclusion at an earlier evidence level therefore did not imply eligibility for a subsequent quantitative level. Thus, the 34 retained reports define the systematic-review corpus, whereas the numbers of reports, contrasts, and independent experimental/publication families contributing to individual pooled outcomes are necessarily outcome-specific. Table 1 reports the number of independent experimental/publication families contributing to each quantitative outcome, with additional model-level information provided in Data S1.

Screening proceeded from deduplicated title and abstract records to full-text eligibility assessment. We searched Scopus and Web of Science on 9 August 2026. The search strategies combined terms related to polyamines, salinity stress, oxidative and redox responses, and plant material. The complete database-specific search strings, including field tags and Boolean operators, are provided in Data S1, Table S10. The searches yielded 1093 records: 638 from Scopus and 455 from Web of Science.

Records from both databases were merged and deduplicated using digital object identifier (DOI) and normalized-title matching. DOI strings were standardized and compared first, followed by normalized title matching for records without a concordant DOI; duplicate records occurring within individual database exports were also checked. The final deduplication was subsequently reproduced computationally using Python 3.12.13 and pandas 2.2.3. This reproducibility audit confirmed the removal of 446 duplicate records, leaving 647 unique records for screening.

One reviewer conducted title and abstract screening and the subsequent full-text eligibility assessment using the prespecified eligibility criteria. To strengthen quality control, a second reviewer retrospectively and independently reassessed a random subset of the screening and eligibility decisions. We resolved any discrepancies by direct comparison with the prespecified eligibility criteria and consensus. This retrospective audit was used as a quality-control procedure and does not replace prospective duplicate screening.

Of the 647 unique records screened, 468 were excluded at the title and abstract stage, and 179 reports were retrieved and assessed for full-text eligibility. Following full-text assessment and reconciliation of record identity, intervention eligibility, and causal contrast structure, 34 reports were retained in the systematic-review corpus. The remaining 145 reports were excluded because they did not meet the prespecified causal eligibility criteria. Item-level reasons for these full-text exclusions were not recoverable from the screening log; therefore, no exclusion-reason subcounts were inferred.

### 2.2. Data Extraction and Outcome Mapping

The frozen working matrix contained 444 complete quantitative contrasts. Extracted information included species and genotype, polyamine identity and dose, application method, salt stressor and concentration, exposure duration, tissue, sample size, treatment means, dispersion statistics, data source, and quantitative quality flags. We organized outcomes into oxidative damage/ROS, antioxidant defense, ion homeostasis, water/osmotic responses, photosynthesis/pigments, growth/biomass, and molecular/metabolic responses. We retained five additional rows as pending because we could not recover complete quantitative inputs. Final small-k inference used the 21 frozen independent-family means provided in Data S1, Table S3 and Supplementary Data S2, SmallK_Family_Inputs.

One reviewer performed the primary data extraction using a standardized extraction framework. To strengthen quality control, a second reviewer retrospectively and independently verified a random subset of the retained studies, including treatment and control values, dispersion measures, sample sizes, polyamine identity and dose, salinity treatment, outcome classification, and experimental family assignment. Extracted quantitative data were subsequently subjected to structured consistency checks and computational verification against the frozen extraction matrix, including validation of treatment and control values, dispersion measures, sample sizes, effect-size calculations, family assignments, and quality-control flags. We performed computational verification and reproducibility checks using Python 3.12.13, pandas 2.2.3, NumPy 2.3.5, SciPy 1.17.0, and openpyxl 3.1.5. Ambiguous or internally inconsistent quantitative information was not resolved by assumption; when a defensible value could not be established from the published report, the corresponding record was retained as pending or excluded from the weighted quantitative synthesis, as appropriate. Study investigators were not contacted for additional data or clarification. This retrospective independent verification was used as a quality-control procedure but did not constitute prospective duplicate extraction of the complete dataset; therefore, residual extraction error or reviewer-related bias cannot be completely excluded.

Extraction was intentionally inclusive within eligible experiments. We retained all eligible polyamine doses, salinity intensities, genotypes, tissues, and sampling times when the required contrast could be reconstructed; no ‘best dose’ was selected. Null and adverse responses were preserved under the same rule. Each publication or experimentally linked set of reports was assigned to an experimental/publication family so that multiple contrasts sharing controls or biological material could be recognized as dependent rather than counted as independent studies.

MDA, H_2_O_2_, and superoxide anion (O_2_•^−^) represented oxidative-damage/ROS outcomes, whereas superoxide dismutase (SOD), catalase (CAT), ascorbate peroxidase (APX), and peroxidase (POD/POX) were retained in their original enzyme-activity direction. Ion homeostasis was harmonized from Na^+^/K^+^ or K^+^/Na^+^ ratios. Relative water content (RWC) was the principal common water-status endpoint. Total chlorophyll was preferred for pigment synthesis when available, and net CO_2_ assimilation (A or Pn) represented photosynthetic performance. For growth, total or whole-seedling dry biomass was preferred; when it was unavailable, shoot dry mass was used as the primary endpoint, while root dry mass was reserved for sensitivity interpretation to avoid double-counting organs from the same experiment.

Numerical values were preferentially taken from tables or explicit text. When numerical values were available only graphically, the primary reviewer extracted treatment means and, where applicable, error bars from high-resolution figures using calibrated plot axes and pixel-to-data coordinate conversion. We flagged figure-derived values during quality control. During revision, we reviewed the figure-derived records against the corresponding study-specific audit trail, and we re-digitized the previously provisional values reported by Li et al. [18] using a Python-based procedure with OpenCV 4.13.0 and NumPy 2.3.5. The revision-stage check found no materially discordant graphical values, so we retained the frozen extraction values. The same reviewer performed all graphical extraction and revision-stage rechecking. When a textual percentage and a plotted value were materially discordant, we did not resolve the conflict by selecting the value that produced the more favorable effect; we withheld the endpoint from the weighted synthesis until we could establish a defensible value. The same conservative principle was applied when error bars could not be unambiguously identified as SD or SE.

### 2.3. Effect Sizes, Variance, and Direction Harmonization

For outcomes in which higher values represented a favorable response, the effect size was calculated as the natural logarithm of the response ratio (lnRR) using Equation (1):

(1) $$lnRR=\ln\left(\frac{{\bar{X}}_{Salt+PA}}{{\bar{X}}_{Salt}}\right)$$

In Equation (1), ${\bar{X}}_{Salt+PA}$ is the mean of the salt-stressed group receiving exogenous polyamine, whereas ${\bar{X}}_{Salt}$ is the corresponding salt-stressed control mean. For direct damage markers in which lower values indicate protection, the sign of the raw lnRR was reversed so that a positive protective lnRR consistently represented lower oxidative burden. This orientation was applied to MDA and terminal H_2_O_2_ accumulation; the underlying raw treatment and control means were retained unchanged in the extraction matrix.

For independent treatment and control groups, the approximate sampling variance of lnRR was calculated using Equation (2):

(2) $$v=\frac{{{SD}_{Salt+PA}}^{2}}{n_{Salt+PA}{{\bar{X}}_{Salt+PA}}^{2}}+\frac{{{SD}_{Salt}}^{2}}{n_{Salt}{{\bar{X}}_{Salt}}^{2}}$$

Equation (2) uses the reported group means, standard deviations (SDs), and sample sizes. When studies reported standard errors (SEs), these were converted to SDs using Equation (3):

(3) $$SD=SE\sqrt{n}$$

No arbitrary variance imputation was used. When a paper supplied a defensible pooled residual variance or analysis-of-variance (ANOVA) mean-square error that could be mapped to the reported group means, we used that information with an explicit quality flag. Mean-only studies remained eligible for the systematic-review and evidence-map layers but were not assigned synthetic sampling variance.

For ionic ratios, Na^+^/K^+^ effects were sign-inverted, whereas K^+^/Na^+^ effects retained their original sign, creating a common favorable orientation without modifying the source measurements. For interpretation on the original proportional scale, favorable higher-is-better outcomes were expressed using Equation (4):

(4) $$100\left[\exp\left(lnRR\right)-1\right]$$

Protective damage effects were expressed using Equation (5):

(5) $$100\left[1-\exp\left(-{lnRR}_{protective}\right)\right]$$

Equations (4) and (5) were used for communication on the proportional scale only; all statistical models were fitted on the lnRR scale.

### 2.4. Statistical Dependence, Family-Aware Quantitative Synthesis, and Evidence Integration

The family-aware framework was designed to address the non-independence created when a single experiment contributes multiple doses, genotypes, tissues, salinity intensities, time points, or treatment comparisons. Here, ‘family’ refers to an experimental/publication family rather than to a botanical taxonomic family. In a conventional synthesis that treats every contrast as independent, experiments reporting many related comparisons can receive disproportionate influence and artificially narrow uncertainty. Our approach instead preserves all eligible contrasts while recognizing their common experimental origin. For outcomes supported by sufficient independent families, we incorporated dependence directly through shared-control covariance and multilevel random effects at the contrast and family levels. For physiological outcomes represented by only four or five independent families, we first reduced all eligible contrasts within each family to one prespecified family mean and then conducted inference across these independent family means. Thus, the framework’s central methodological feature is that the experimental/publication family, rather than the individual contrast, defines the effective unit of independent evidence, allowing multiple treatment conditions to be retained without treating them as independent replications.

The primary unit of independence was the experimental/publication family. Multiple doses, genotypes, tissues, salinity intensities, or time points from a common experiment were retained but linked through dependency-cluster and publication-family identifiers. For comparisons sharing a salt-stressed control, the off-diagonal sampling covariance was estimated from the control contribution using Equation (6):

(6) $${Cov}_{ij}=\frac{{{SD}_{Salt}}^{2}}{n_{Salt}{{\bar{X}}_{Salt}}^{2}}$$

The covariance in Equation (6) was capped at $0.999\sqrt{v_{i}v_{j}}$ only when rounding or digitization would otherwise imply an invalid correlation. This sampling covariance was retained in every multilevel fit.

For MDA, H_2_O_2_, O_2_•^−^, SOD, CAT, APX, and POD/POX, publication-family-aware multilevel random-effects models were specified using Equation (7):

(7) $$V=S+\tau_{within}^{2}I+\tau_{family}^{2}Z_{family}{Z_{family}}^{\prime }$$

In Equation (7), S is the sampling covariance matrix, I is the identity matrix associated with contrast-level residual heterogeneity, and $Z_{family}$ is the design matrix identifying the experimental/publication family. We estimated the two variance components by restricted maximum likelihood (REML) and the pooled intercept by generalized least squares. Two-sided 95% confidence intervals used the normal approximation. MDA and H_2_O_2_ were designated principal oxidative outcomes; O_2_•^−^ was interpreted as exploratory because only five independent families were available and its confidence interval was close to the null. Leave-one-family-out refits assessed robustness of the principal oxidative estimates.

Polyamine identity was evaluated only as an exploratory moderator for MDA and H_2_O_2_. Spermidine was the reference category, with indicator terms for putrescine and spermine; the same shared-control covariance and two-component random-effects structure were retained. Overall moderation was evaluated with a two-degree-of-freedom Wald χ^2^ test. A compound was not declared superior merely because its predicted subgroup mean was numerically larger. After all, category sizes were unequal and compound identity was confounded with dose, species, stress severity, and experimental design.

For physiological outcomes represented by only four or five independent families, contrast-level cluster-robust inference was considered too unstable for the primary estimate. All eligible lnRR values within an experimental/publication family were therefore aggregated to one frozen family mean, without selecting a ‘best’ dose or treatment condition. The independent family means were summarized by an unweighted mean lnRR, between-family SD, and a two-sided Student’s t interval with df = k − 1. Leave-one-family-out means were retained as a sensitivity diagnostic. This deliberately conservative strategy protects the family as the inferential unit, although it does not recover the within-study precision weighting possible with more independent clusters.

The systematic-review layer retained biologically eligible studies even when a weighted effect size could not be estimated for a particular endpoint. Study-level evidence was mapped across seven domains—oxidative damage/ROS, antioxidant defense, ion homeostasis, water/osmotic responses, photosynthesis/pigments, growth/biomass, and molecular/metabolic responses—and was also classified by quantitative readiness. The mechanistic synthesis then integrated the direction and certainty of the quantitative results with recurrent signaling, metabolic, ionic, water-relations, photosynthetic, and growth processes reported across the retained literature. This evidence hierarchy distinguished quantitatively reproducible responses from mechanistically plausible but less consistently reported pathways.

All final effect-size definitions, orientation rules, family assignments, small-k inputs, sensitivity diagnostics, and quality-control flags were frozen in the project workbooks before preparation of the revised submission package. Analyses were executed in Python 3.12.13 using NumPy 2.3.5, SciPy 1.17.0, pandas 2.2.3, and openpyxl 3.1.5. The supplied script independently recalculates all 444 complete lnRR and variance fields, refits the seven multilevel models, performs the moderator and leave-one-family-out analyses, and reproduces the five small-k syntheses. Formal funnel-plot or asymmetry tests were not performed because independent-family counts and nested dependence were too limited for interpretable assessment. We did not apply a formal certainty-grading framework or study-level risk-of-bias instrument because the retained literature encompassed heterogeneous experimental designs, reporting structures, and physiological endpoints for which no single standard instrument could be applied consistently without substantial subjective adaptation. Confidence was therefore interpreted descriptively based on family count, precision, dependence control, consistency, and sensitivity. Numerical readiness and source auditing were used for quality control but were not substitutes for a formal assessment of study-level internal validity; this absence is treated as a methodological limitation.

## 3. Results

### 3.1. Study Selection, Evidence Landscape, and Quantitative Readiness

**Figure 1:**
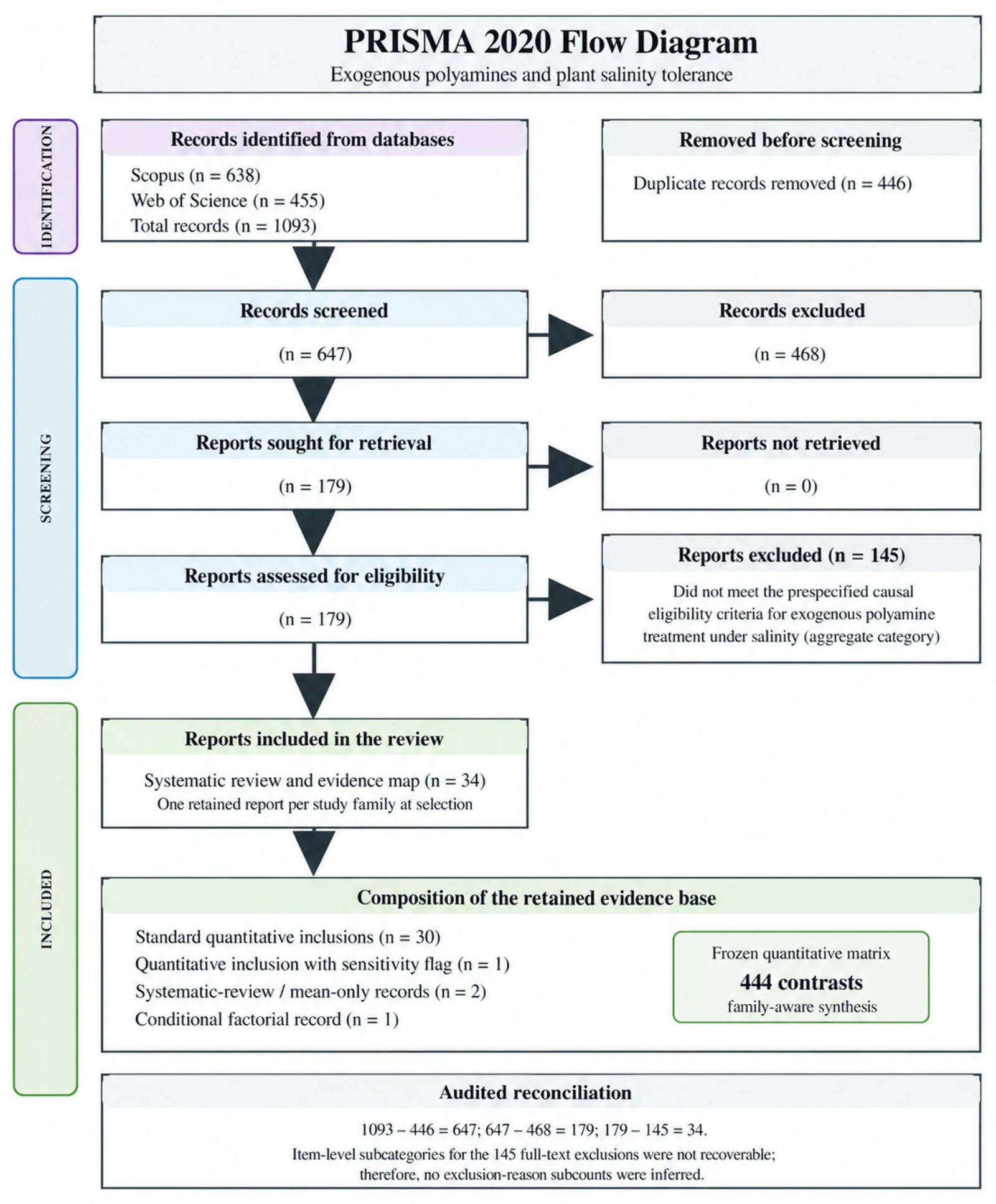
Preferred Reporting Items for Systematic Reviews and Meta-Analyses (PRISMA) 2020 flow diagram for study identification, screening, eligibility, and inclusion. The 145 full-text exclusions are reported as an audited aggregate because the supplied screening log did not include item-level exclusion categories; no reason subcounts were inferred.

The database searches identified 1093 records. After removing 446 duplicates, 647 unique records were screened, and 468 were excluded at title/abstract screening, leaving 179 reports for full-text eligibility assessment; all 179 were retrieved. Of these 179 full-text reports, 145 were excluded, and 34 were retained in the systematic-review corpus. The screening log preserved the aggregate number of full-text exclusions but not report-level exclusion reasons; therefore, reliable category-specific exclusion counts could not be reconstructed without retrospective inference. Consider this limitation when interpreting the transparency of the study-selection process. The Study_Screening matrix contains 35 fully characterized records: 34 were retained in the systematic-review/evidence-map corpus, and one was excluded because the exogenous intervention was not a polyamine. The 34 retained reports comprise 30 standard quantitative inclusions, one quantitative inclusion retained with an explicit sensitivity flag, two systematic-review/mean-only records, and one conditional factorial study [2,3,6,7,18,19,20,21,22,23,24,25,26,27,28,29,30,31,32,33,34,35,36,37,38,39,40,41,42,43,44,45,46,47]. The study-selection process is summarized in Figure 1.

**Figure 2:**
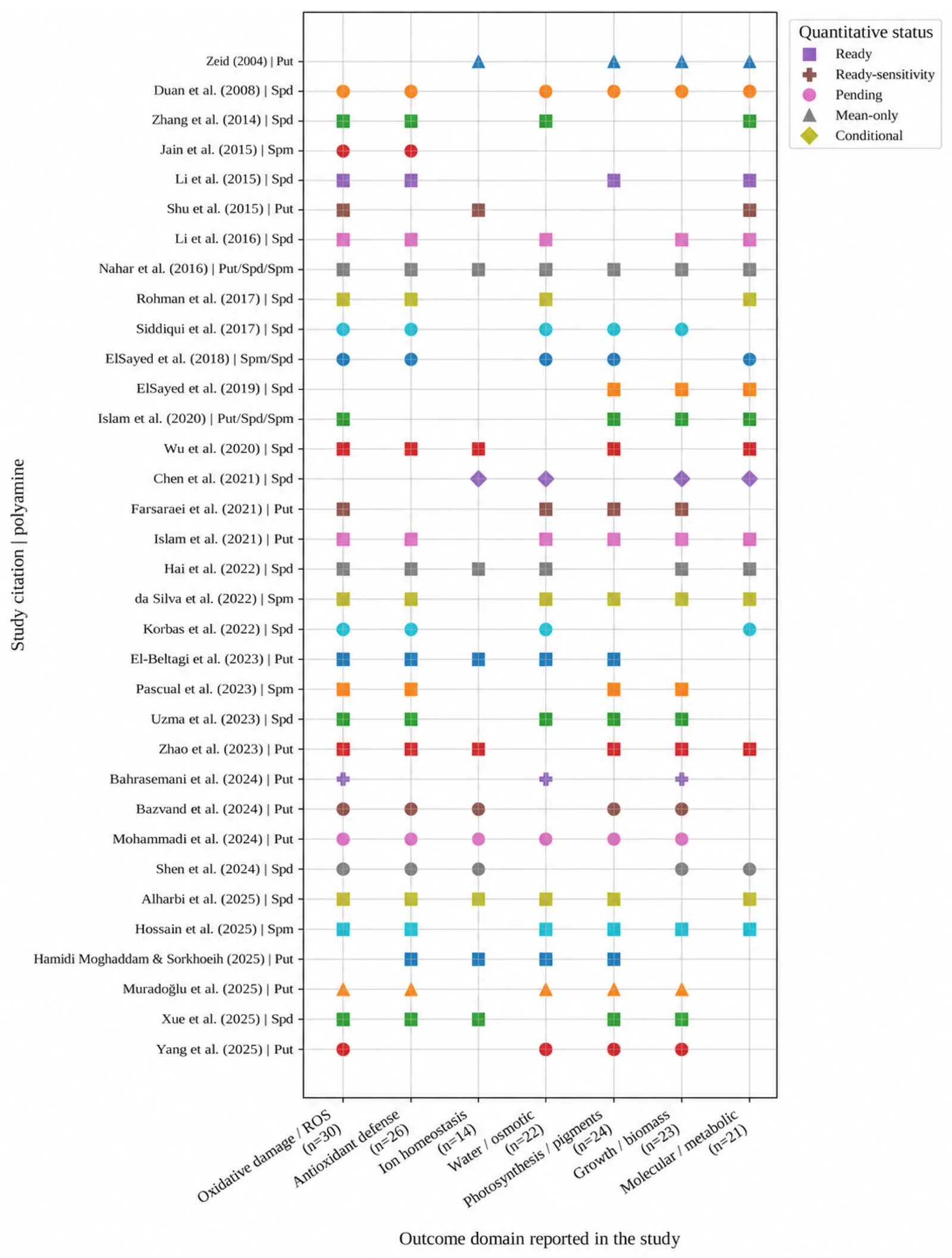
Evidence map of exogenous polyamine studies under salinity stress. Study labels use first-author and publication-year format (et al. for multi-author reports, where applicable) and correspond to the References and Table S1. Symbols indicate the quantitative status and identify whether an outcome domain was reported in each retained study; colors are used only to facilitate visual distinction among study rows. The presence of a symbol does not imply that the study contributed a weighted effect to every quantitative model [2,3,6,7,18,19,20,21,22,23,24,25,26,27,28,29,30,31,32,33,34,35,36,37,38,39,40,41,42,43,44,45,46,47].

The evidence map shows a field strongly centered on redox biology: 30 of the 34 retained records reported oxidative-damage or ROS-related outcomes and 26 assessed antioxidant-defense responses. However, the literature also covered photosynthesis or pigments (24 studies), growth or biomass (23), water or osmotic responses (22), molecular or metabolic processes (21), and ion homeostasis (14). This breadth supports an integrated physiological synthesis rather than a review restricted to antioxidant biomarkers. For direct traceability to the source literature, Figure 2 identifies each retained report using first-author citation format, while Table S1 preserves the study-level evidence classification.

The distribution of polyamine treatments was also uneven. Spermidine was the most frequently represented single polyamine in the retained evidence map (15 studies), followed by putrescine (12) and spermine (4). Two studies evaluated Put, Spd, and Spm within the same experimental framework, and one additional study compared Spm and Spd. Quantitative readiness did not perfectly overlap with biological eligibility: 21 records were directly ready for quantitative synthesis, one was usable with a prespecified sensitivity flag, nine remained eligible but contained unresolved numeric-recovery or dispersion issues, two contributed mean-only/systematic-review evidence, and one had a conditional factorial structure. 

### 3.2. Oxidative Damage and Antioxidant-Defense Responses

The strongest cross-study oxidative signal was reduced damage under salinity, particularly for MDA and H_2_O_2_. For malondialdehyde (MDA), the publication-family-aware multilevel model included 15 independent families, 16 publications, and 61 contrasts. The pooled protective lnRR was 0.214 (95% CI 0.138–0.289), corresponding to an estimated 19.2% reduction in MDA (95% CI 12.9–25.1%). Estimated heterogeneity was τ^2^within = 0.0179 and τ^2^family = 0.0132. The direction was stable in leave-one-family-out analyses, with estimated reductions ranging from 17.0% to 20.7%.

**Figure 3:**
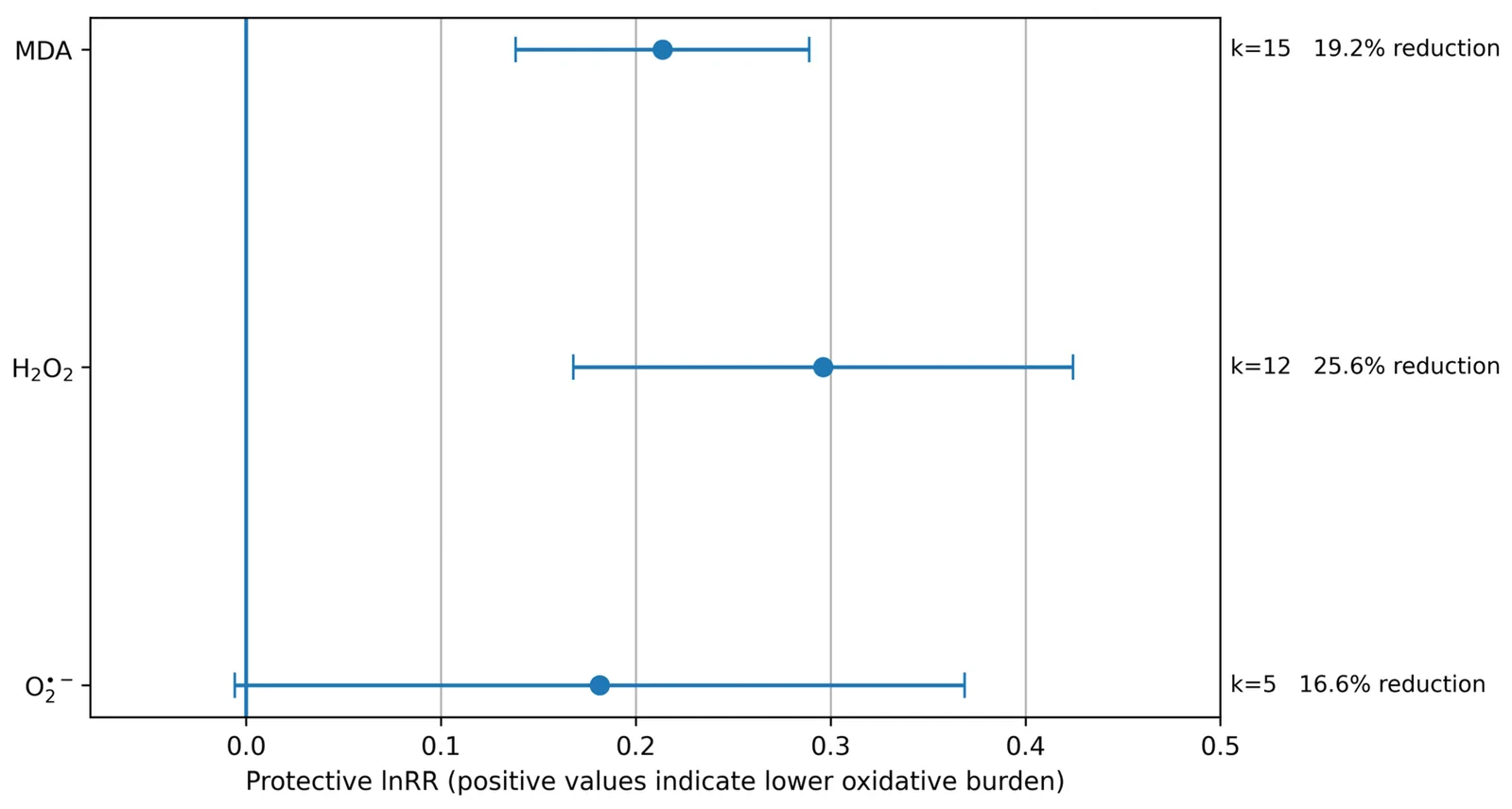
Summary of pooled effects of exogenous polyamines on oxidative-damage markers under salinity. Positive protective lnRR values indicate a reduction in oxidative burden relative to the corresponding salt-stressed control. The vertical line at lnRR = 0 represents the null-effect reference. MDA and H_2_O_2_ are final publication-family-aware multilevel estimates; O_2_•^−^ remains exploratory. Abbreviations: H_2_O_2_, hydrogen peroxide; lnRR, natural logarithm of the response ratio; MDA, malondialdehyde; O_2_•^−^, superoxide anion.

H_2_O_2_ showed an even larger pooled response: 12 independent families and 41 contrasts yielded a protective lnRR of 0.296 (95% CI 0.168–0.424), equivalent to a 25.6% reduction (95% CI 15.5–34.6%), with τ^2^within = 0.0164 and τ^2^family = 0.0400. Leave-one-family-out estimates ranged from 23.2% to 28.3% reduction. By contrast, superoxide (O_2_•^−^) was less certain. Across five independent families, six publications, and 18 contrasts, the protective lnRR was 0.181 (95% CI −0.006 to 0.369), with τ^2^within = 0.0357 and τ^2^family = 0.0310; the interval narrowly included zero. The small number of independent families (k = 5) likely contributed to the limited precision of this estimate and therefore warrants cautious interpretation. The pooled oxidative-damage estimates are summarized in Figure 3.

Antioxidant–enzyme responses were more variable. Pooled lnRR values were 0.145 for SOD (six families, 21 contrasts), 0.120 for CAT (seven families, 23 contrasts), 0.110 for APX (seven families, 18 contrasts), and 0.079 for POD/POX (five families, 18 contrasts), corresponding to approximate activity changes of +15.6%, +12.8%, +11.6%, and +8.2%, respectively. For SOD, CAT, APX, and POD/POX, τ^2^ within was 0.0229, 0.0143, 0.0063, and 0.0763, while τ^2^family was 0.0285, 0.0239, 0.0307, and 0.0035, respectively. Every 95% confidence interval crossed zero, indicating substantial context dependence and supporting joint interpretation of enzyme activity with direct damage markers.

**Figure 4:**
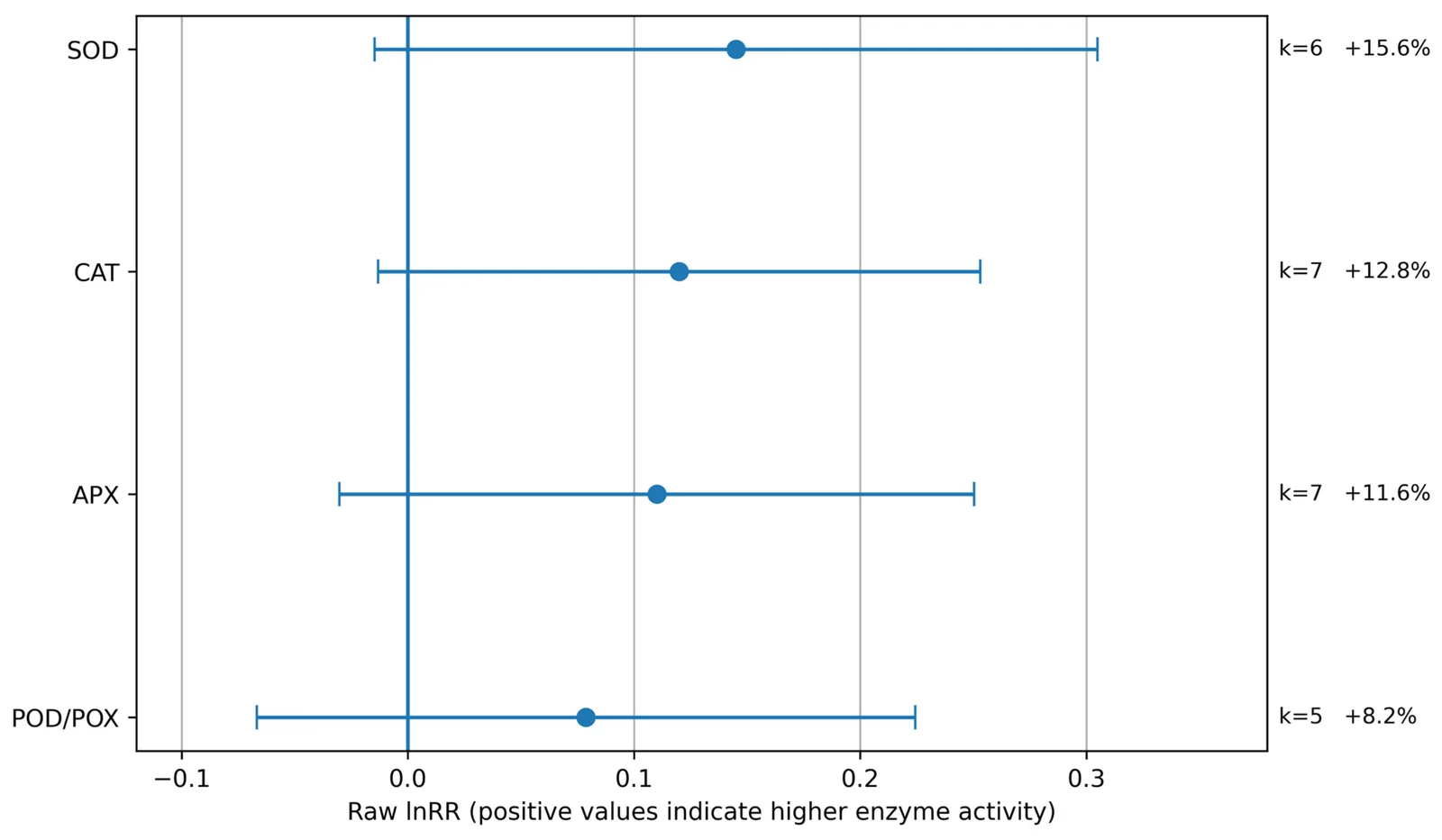
Antioxidant–enzyme responses to exogenous polyamines under salinity. Raw lnRR values are shown; positive values indicate higher enzyme activity. The vertical line at lnRR = 0 represents the null-effect reference. Enzyme activity was not sign-flipped into a generic protection score. Abbreviations: APX, ascorbate peroxidase; CAT, catalase; lnRR, natural logarithm of the response ratio; POD/POX, peroxidase; SOD, superoxide dismutase.

Taken together, the redox results separated two different levels of response. Direct damage markers provided the clearest cross-study signal: the confidence intervals for MDA and H_2_O_2_ excluded the null and remained stable in family-level sensitivity analyses, whereas the O_2_•^−^ estimate was less precise. In contrast, none of the four major antioxidant-enzyme pools showed a cross-family confidence interval clearly separated from zero. The evidence therefore supports a consistent reduction in net oxidative burden without requiring a uniform increase in any single antioxidant enzyme. The antioxidant–enzyme estimates are summarized in Figure 4.

### 3.3. Ion–Water Homeostasis, Photosynthetic Performance, and Biomass

Ion balance showed a favorable response across five independent experimental/publication families. The conservative family-level mean lnRR was 0.549 (between-family SD = 0.280; 95% CI 0.201–0.896), corresponding to an equivalent 73.1% improvement in the favorable K^+^/Na^+^ orientation (95% CI 22.3–145.0%). The large interval indicates substantial between-family variation in magnitude, but the direction remained favorable after omission of each family in turn. Nevertheless, because this estimate is based on only five independent families, its magnitude should be regarded as preliminary and interpreted cautiously. Chen et al. [31] was retained as conditional/sensitivity evidence because the spermidine effect could be isolated within endophyte strata. However, ratio-level dispersion was not sufficiently precise for the primary synthesis.

Relative water content showed a favorable response across four independent families. The family-level mean lnRR was 0.123 (between-family SD = 0.074; 95% CI 0.005–0.241), equivalent to an approximately 13.0% increase in RWC (95% CI 0.5–27.2%). The lower confidence bound was close to the null, and with only four independent families, this estimate should be considered preliminary, supporting a modest rather than strong effect. Together, the ion-balance and RWC syntheses support ionic and water-status stabilization as a recurrent component of polyamine-mediated salinity tolerance.

Total chlorophyll showed a favorable mean response across four independent families, but uncertainty remained substantial. The mean lnRR was 0.230 (between-family SD = 0.160; 95% CI −0.025 to 0.485), equivalent to +25.9% (95% interval −2.4% to +62.4%). Net photosynthetic rate (A/Pn) was more heterogeneous: the mean lnRR was 0.144 across four families (between-family SD = 0.409; 95% CI −0.507 to 0.795), equivalent to +15.5% on average but compatible with changes from −39.8% to +121.5%. The *Tropaeolum majus* spermine family illustrates an important source of this heterogeneity. In this factorial experiment, 1 mM Spm was evaluated under moderate and severe NaCl stress, but the A/Pn response was not uniformly favorable across salinity intensities. In particular, the severe-salinity contrast showed a strongly adverse photosynthetic response and was retained rather than excluded as an outlier, despite beneficial responses in other physiological traits [34]. Thus, chlorophyll preservation remains suggestive, and there is no evidence for a consistent universal rescue of carbon assimilation. Both estimates are also preliminary because each is supported by only four independent families.

Dry biomass showed a favorable response across four independent families. The mean lnRR was 0.382 (between-family SD = 0.133; 95% CI 0.171–0.594), corresponding to an approximately 46.6% increase in dry biomass (95% CI 18.6–81.1%). The direction remained positive in leave-one-family-out analyses, indicating that the result was not dependent on a single family, although the estimate should remain preliminary because it is based on only four independent families.

**Figure 5:**
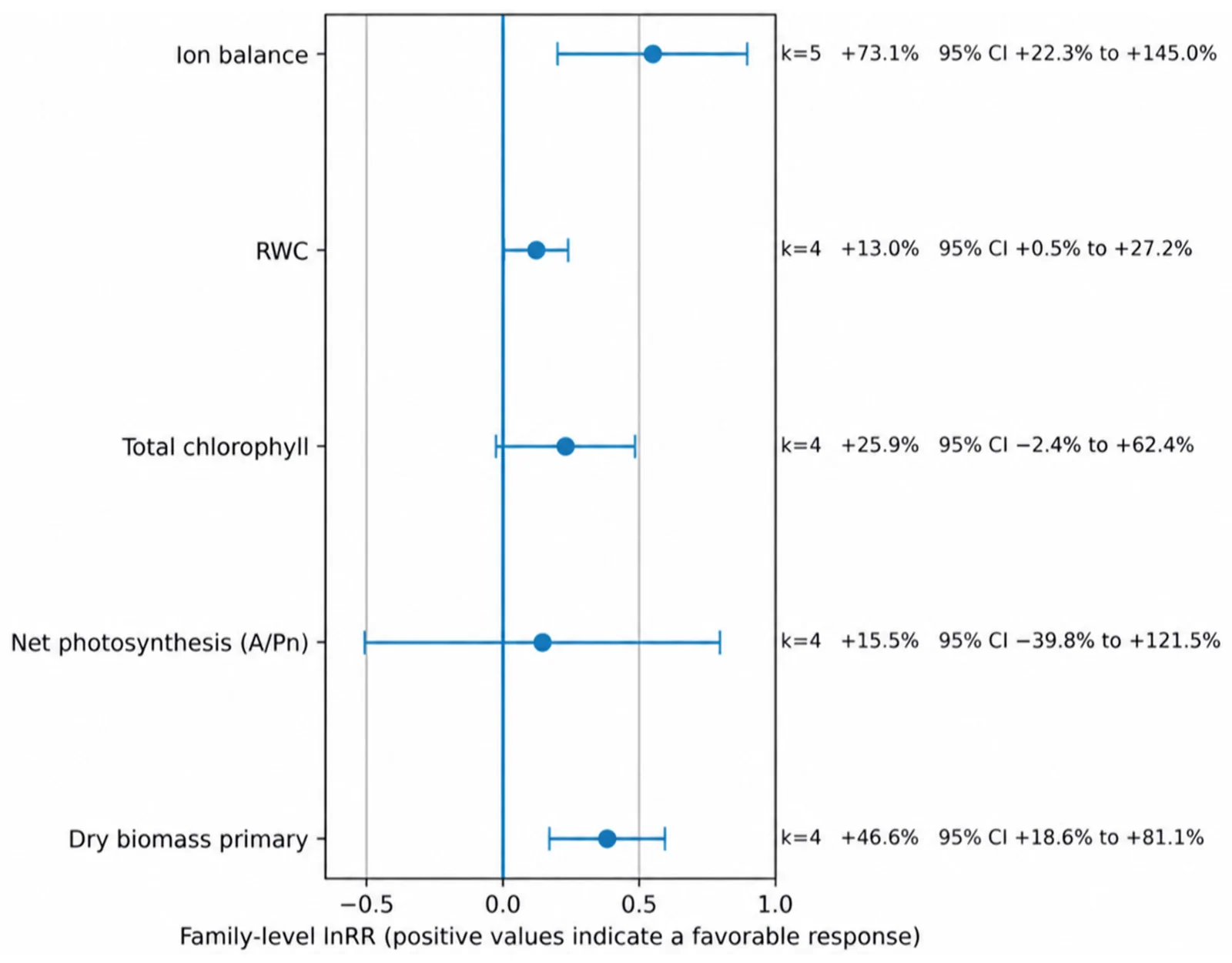
Conservative independent-family synthesis of physiological responses to exogenous polyamines under salinity. Each experimental/publication family contributes one aggregated lnRR, preventing multiple doses, genotypes, tissues, or shared controls from being treated as independent studies. Points show the mean family-level lnRR and horizontal bars show 95% Student’s *t* intervals (df = k − 1). The vertical line at lnRR = 0 represents the null-effect reference. Positive values indicate a favorable response. Abbreviations: A/Pn, net CO_2_ assimilation/net photosynthetic rate; CI, confidence interval; df, degrees of freedom; lnRR, natural logarithm of the response ratio; RWC, relative water content. *k* indicates the number of independent experimental/publication families.

Across these downstream physiological traits, the strength of evidence followed a clear gradient. Ion balance and dry biomass retained confidence intervals above the null in the conservative independent-family synthesis. At the same time, RWC showed a smaller positive effect with its lower bound close to zero. Total chlorophyll was favorable on average but remained statistically uncertain, and A/Pn was compatible with both substantial benefit and substantial reduction. Thus, the physiological synthesis did not support a single uniform ‘stress recovery’ response; rather, homeostatic traits were more reproducible across study families than instantaneous carbon assimilation. The independent-family physiological estimates are summarized in Figure 5.

Descriptive exploration suggested that heterogeneity was not attributable to polyamine identity alone. For MDA, the estimated reductions were 23.1% for Put, 18.4% for Spd, and 18.7% for Spm, whereas the corresponding values for H_2_O_2_ were 24.6%, 19.8%, and 26.9%, respectively; these subgroup estimates were exploratory, and formal PA-type moderator tests did not identify clear between-polyamine differences. Variation was greater across experimental contexts. Among the small-k physiological syntheses, independent-family responses ranged from approximately 16.8% to 117.7% for ion balance, 3.7% to 20.5% for RWC, 3.6% to 52.0% for total chlorophyll, −36.0% to +61.1% for A/Pn, and 29.3% to 74.9% for dry biomass. Within individual experiments, responses also varied with dose and salinity intensity, including stronger effects at intermediate polyamine doses and an adverse A/Pn response under severe salinity in the *Tropaeolum majus* spermine family. Species or genotype, application method, tissue, stress intensity, dose, and exposure duration were strongly confounded across the available studies and were too sparsely represented for reliable independent moderator testing.

### 3.4. Integrated Quantitative Pattern

Table 1 summarizes the overall quantitative evidence. Reductions in oxidative damage, particularly MDA and H_2_O_2_, were the most consistent cross-family responses. Ion balance and dry biomass also showed favorable mean effects. However, these and the other physiological estimates were based on only four or five independent families and should therefore be considered preliminary. RWC showed a more modest positive response, whereas antioxidant–enzyme activities, total chlorophyll, and especially net photosynthesis were more variable or uncertain. Overall, the evidence was strongest for attenuation of oxidative damage and progressively less consistent across downstream physiological outcomes.

## 4. Discussion

### 4.1. Redox Regulation as the Primary Mechanistic Axis

The quantitative and systematic-review layers converge on a hierarchical interpretation of polyamine-mediated salinity tolerance. The most reproducible signal across the evidence base was attenuation of oxidative damage. MDA and H_2_O_2_ were the only outcomes supported by comparatively large numbers of independent families and final multilevel models whose confidence intervals clearly excluded the null. This matters because much of the experimental polyamine literature has traditionally described tolerance through the induction of SOD, CAT, APX, or peroxidases. Our synthesis suggests that this enzyme-centered view is incomplete. Across species and experimental systems, the more transferable result is that exogenous polyamines are associated with a lower final oxidative burden, whereas the particular enzymatic route used to achieve that state varies [6,18,37,44].

The enzyme results reinforce this interpretation. SOD, CAT, APX, and POD/POX all had positive average raw lnRR values, but every confidence interval crossed zero. A larger enzyme activity therefore cannot be treated as a universal marker of successful protection. Enzyme activity may rise because a polyamine treatment primes antioxidant capacity before or during stress. However, it may also decline after ROS production has been reduced and detoxification demand has fallen. The same final phenotype—less membrane damage—can therefore emerge from different combinations of ROS production, enzymatic detoxification, non-enzymatic antioxidant pools, and repair. For interpretation, read direct damage markers and enzyme activities together rather than arranging them in a simple causal chain in which more enzyme activity automatically means more tolerance.

A second implication is that H_2_O_2_ itself has to be interpreted temporally. Polyamine oxidation can generate a localized H_2_O_2_ signal that precedes acclimation, while prolonged H_2_O_2_ accumulation at later sampling points is more consistent with unresolved oxidative pressure. In tobacco, apoplastic spermidine oxidation generated H_2_O_2_ signatures linked to stress acclimation [10]. In tomato, both PAO-dependent and RBOH-dependent H_2_O_2_ pathways have been implicated in Spd- or Spm-induced activation of antioxidant defenses under saline or saline–alkaline conditions [11,48]. Cross-context genetic evidence also shows that putrescine can amplify RBOHD/F-dependent H_2_O_2_ signaling in *Arabidopsis* [49]. These findings explain why the meta-analytic reduction in final H_2_O_2_ should not be interpreted as evidence that H_2_O_2_ signaling is undesirable; instead, the relevant distinction is between transient signaling fluxes and sustained oxidative burden. This temporal distinction may also have a spatial counterpart. Because polyamine effects on K^+^ retention differ markedly between mature and elongating root zones [9], localized polyamine-dependent H_2_O_2_ signaling may contribute to tissue-specific regulation of ion fluxes and tolerance. This connection remains mechanistically plausible but is not directly demonstrated by the present quantitative synthesis.

The interaction with nitric oxide adds another layer to this temporal model. The rapid induction of NO by Spd and Spm in *Arabidopsis* demonstrates that polyamines can alter nitrosative signaling independently of later antioxidant outcomes [12]. In salinity-exposed citrus, polyamine treatment altered both oxidative and nitrosative signatures and reorganized the proteomic response, including proteins whose abundance or redox-related modification changed under stress [13]. This broader evidence is consistent with the meta-analytic result that no single antioxidant enzyme can explain the reduction in oxidative burden. The relevant phenotype is likely generated by coordinated control of ROS production, ROS removal, NO-related signaling, membrane stability, metabolism, and protein-level acclimation.

### 4.2. From Ion–Water Homeostasis to Photosynthetic Performance and Growth

The final family-level syntheses suggest ion and water homeostasis as a second recurrent axis of the response. However, these physiological estimates remain preliminary because only four or five independent families support them. Ion balance remained favorable across all five independent families, and the confidence interval stayed above the null despite the large variation in effect magnitude. RWC showed a smaller but still positive mean effect. These outcomes are physiologically coherent because salinity tolerance depends not only on excluding or compartmentalizing Na^+^ but also on retaining K^+^, preserving membrane selectivity, and maintaining enough cellular water to sustain metabolism. Polyamines can influence these processes at the membrane level; for example, they have been shown to reduce salt-induced K^+^ efflux through non-selective cation channels, while long-term spermine responses in *Arabidopsis* also support a role in ionic acclimation [1,15]. The present synthesis therefore links the redox and ionic components of tolerance rather than treating them as independent mechanisms.

The water-status signal is also broader than RWC alone. Polyamine treatments frequently modify osmotic adjustment, compatible solutes, nutrient balance, and carbonyl detoxification, but these variables were too heterogeneous in definition and reporting to support a common meta-analytic endpoint. In mung bean, exogenous Put, Spd, and Spm improved nutrient homeostasis while strengthening antioxidant defense and the glyoxalase system, linking salt tolerance not only to ROS control but also to detoxification of methylglyoxal generated during metabolic stress [6]. In tomato, exogenous Spd altered endogenous polyamine metabolism under salinity–alkalinity stress, illustrating that the applied compound becomes part of a dynamic metabolic network rather than remaining an external protective molecule [5]. The systematic-review evidence therefore supports a broader homeostatic interpretation even where a standardized effect size could not be calculated.

Ion-homeostasis responses are also tissue-dependent. Identical polyamine treatments can preserve K^+^ in mature root zones while intensifying NaCl-induced K^+^ loss in elongating tissues [9], providing one plausible source of the between-family variation observed in the ion-balance synthesis.

Molecular evidence also provides a direct bridge between Spd metabolism and Na^+^/K^+^ regulation. In tomato, SlWRKY81 was shown to bind the promoters of SlSPDS2 and SlNHX4 under saline–alkali stress; activation of this module increased Spd content, enhanced K^+^ absorption, and promoted Na^+^ efflux, while jasmonate signaling modulated the pathway through SlJAZ1 [14]. This result is particularly relevant to the present synthesis because it connects three components that emerge independently from the review—polyamine metabolism, hormonal signaling, and ion homeostasis—within a single genetically supported pathway.

The downstream photosynthetic results were much less uniform. Total chlorophyll was favorable on average, but its independent-family confidence interval included the null, and the A/Pn interval was exceptionally wide. This divergence indicates that preserving pigments or reducing oxidative damage is not equivalent to restoring carbon assimilation. Photosynthesis under salinity remains limited by stomatal closure, mesophyll conductance, ion toxicity, biochemical constraints on the Calvin cycle, altered source-sink balance, and the energetic cost of maintaining ion gradients. Consequently, a plant may exhibit lower MDA, better K^+^/Na^+^ status, and greener leaves while still operating at a restricted photosynthetic rate. The adverse A/Pn contrasts retained in the synthesis are therefore informative rather than anomalous: they prevent the mechanism from being simplified into a universal photosynthetic rescue [2,3,28,37].

The nasturtium studies illustrate why this heterogeneity should be preserved rather than smoothed away. In one factorial experiment, 1 mM Spm was evaluated under both moderate and severe NaCl stress, and the authors concluded that spermine reduced several harmful effects of salinity across growth, gas exchange, water status, lipid peroxidation, and antioxidant responses [34]. However, our contrast-level extraction showed that the direction of A/Pn was not uniformly beneficial across salt intensities; the severe-salinity contrast was strongly adverse even though other traits improved. A separate nasturtium experiment comparing Put, Spd, and Spm likewise reported that mitigation of gas exchange and growth was more evident under moderate stress and depended on both the polyamine and the trait [50]. These examples show that a treatment can be physiologically beneficial at the whole-experiment level while remaining neutral or harmful for a specific downstream endpoint. For that reason, the present synthesis retains adverse contrasts and avoids reclassifying them based on the authors’ overall narrative conclusion.

Biomass provides a longer-term integrative endpoint and, unlike A/Pn, shows a favorable mean response across four independent families; however, the small number of families means that this estimate should be regarded as preliminary. This does not imply that growth is affected only through photosynthesis. Biomass integrates cumulative carbon gain with leaf expansion, respiratory costs, osmotic adjustment, root function, nutrient acquisition, tissue turnover, and the energetic expense of defense. A treatment that reduces oxidative and ionic injury may therefore improve final biomass even when instantaneous A/Pn measured at one sampling point is only weakly affected. This temporal integration is especially important in studies in which early measurements are close to the salt control, but treatment differences become larger after continued exposure [3,28,44].

### 4.3. Context Dependence: Polyamine Identity, Dose, Stress Severity, and Duration

The evidence base includes Put, Spd, and Spm, but it does not support a universal ranking among them. Spermidine is the most frequently represented single polyamine in the retained corpus, while spermine has substantially fewer independent studies. Direct within-experiment comparisons are also uncommon. Under these conditions, an apparent difference among pooled subgroup means can reflect species composition, dose range, stress severity, or publication structure as easily as intrinsic biochemical superiority. Consistent with this limitation, exploratory PA-type moderator tests for MDA and H_2_O_2_ did not show clear between-polyamine differences. The defensible conclusion is therefore not that the three compounds are equivalent, but that current data are insufficient to identify a generally superior polyamine [6,11,15,27].

The historical literature also cautions against using endogenous accumulation as a surrogate for effectiveness. In rice, spermine accumulated during prolonged salinity in both a tolerant and a sensitive cultivar, and pharmacologically increasing spermine did not reduce leaf injury [8]. Conversely, spermine-deficient *Arabidopsis* mutants are hypersensitive to high salinity and can be rescued by exogenous Spm [4]. These apparently contrasting observations are not mutually exclusive: they indicate that the physiological role of a polyamine depends on whether its endogenous pool is limiting, where it accumulates, how rapidly it is metabolized, and which downstream transport or signaling components are competent to respond.

Biological differences among Put, Spd, and Spm are still likely to matter because they differ in charge density, compartmentation, metabolism, transport, and interactions with H_2_O_2_ and NO signaling. However, these properties interact strongly with dose and tissue context. Exogenous Spd can modify endogenous polyamine turnover [20], concentration-dependent responses occur within the same species [24], and identical polyamine treatments can produce opposite ion-flux responses in distinct root zones [9]. Thus, treatment effects are better viewed as polyamine-by-dose-by-tissue-by-environment interactions than as fixed properties of individual compounds.

Hormonal cross-talk is another source of context dependence. In Zinnia, exogenous Put was associated with salt-stress mitigation together with changes in antioxidant activity, K^+^ uptake, and the abscisic acid (ABA)-to-gibberellin balance [41]. In tomato, the genetically resolved SlWRKY81-SlSPDS2-SlNHX4 module operates within a jasmonate-dependent regulatory framework, directly connecting Spd synthesis with Na^+^/K^+^ homeostasis and hormonal signaling [14]. These observations make it unlikely that a single PA concentration has a fixed effect across developmental stages or stress histories, because the response depends on the tissue’s hormonal state and signaling competence at the time of application.

This interpretation also changes how future moderator analyses should be designed. Rather than testing PA identity in isolation, adequately powered datasets should evaluate compound identity together with dose, application route, salt concentration, stress duration, species or genotype, and developmental stage. The breadth of context in the retained literature is illustrated by putrescine studies in ornamental and medicinal species such as Zinnia and henna, where treatment responses were evaluated together with ionic, hormonal, growth, or biochemical traits [41,45]. Direct factorial comparisons among Put, Spd, and Spm within the same biological system would be particularly valuable because they would separate compound-specific effects from study-to-study heterogeneity. Until such evidence accumulates, the present review treats PA identity as exploratory and places greater weight on the reproducibility of the broader redox-homeostasis response.

### 4.4. Integrated Evidence and Research Priorities

The quantitative models answer only part of the research question, so the systematic review and evidence map layers remain central to the article. Several mechanistically informative studies could not contribute a weighted effect size for every trait because they lacked dispersion statistics, used incompatible endpoints, or employed factorial designs that complicated isolating a single contrast. Excluding those studies from the narrative synthesis would bias the biological picture toward the subset of experiments that happened to report quantitative-synthesis-ready statistics. Conversely, including them in weighted models through imputed or guessed variances would create false precision. The integrated design therefore allows different evidence types to contribute at the level they can support: weighted effects for quantitatively defensible contrasts, evidence-map coverage for eligible but incompletely reported studies, and mechanistic interpretation for experimentally informative pathways.

Several features strengthen the quantitative component. The extraction preserved adverse and null responses rather than selecting the most favorable dose; multiple doses, genotypes, tissues, and time points were kept but modeled within experimental/publication families; Na^+^/K^+^ and K^+^/Na^+^ were harmonized only at the analytic effect-size level while the original measurements were retained; enzyme activity was not sign-flipped into an artificial ‘protection’ score; and no arbitrary variance was assigned to studies lacking defensible dispersion. These choices reduce the risk that the synthesis reproduces the direction implied by the review question.

Retaining counterexamples is also important for the credibility of the mechanistic model. A synthesis that included only treatment combinations that improved MDA, A/Pn, or biomass would almost inevitably produce a simple positive narrative. Here, adverse or null responses were preserved when they satisfied the prespecified causal contrast, including the severe-salinity photosynthetic response in nasturtium and the unfavorable ion-balance contrast at the lower salinity level in henna. Their presence changes the interpretation of the pooled evidence: polyamines are not general-purpose antidotes to salinity, and downstream benefits cannot be assumed from biochemical protection alone. The most defensible conclusion is therefore probabilistic and hierarchical—polyamine treatment increases the likelihood of redox and homeostatic stabilization. In contrast, translating that stabilization into photosynthetic or growth recovery remains conditional.

**Figure 6:**
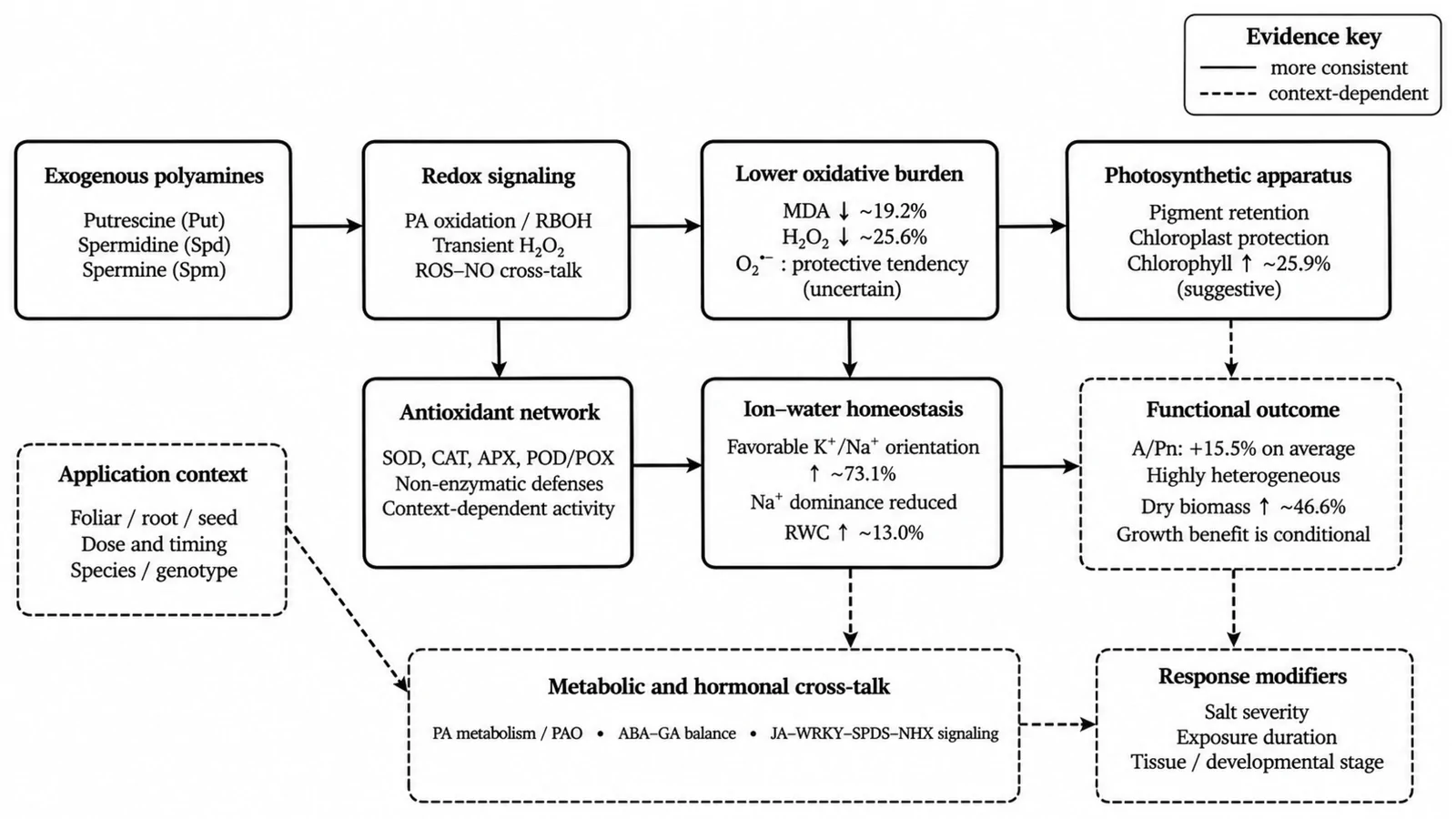
Integrated evidence framework of exogenous polyamine-mediated salinity tolerance in plants. The diagram integrates the evidence hierarchy emerging from the systematic review and quantitative synthesis. Solid arrows indicate more consistently supported relationships, whereas dashed arrows denote context-dependent relationships. Quantitative values shown in the diagram represent the principal publication-family-aware estimates relative to the corresponding salt-stressed controls. Application context, metabolic and hormonal cross-talk, and experimental conditions shape how redox and ion–water stabilization translate into downstream functional outcomes. Abbreviations: PA, polyamine; Put, putrescine; Spd, spermidine; Spm, spermine; RBOH, respiratory burst oxidase homolog; ROS, reactive oxygen species; NO, nitric oxide; MDA, malondialdehyde; H_2_O_2_, hydrogen peroxide; O_2_•^−^, superoxide anion; SOD, superoxide dismutase; CAT, catalase; APX, ascorbate peroxidase; POD/POX, peroxidase; K^+^, potassium; Na^+^, sodium; RWC, relative water content; A/Pn, net CO_2_ assimilation/net photosynthetic rate; PAO, polyamine oxidase; ABA, abscisic acid; GA, gibberellin; JA, jasmonic acid; WRKY, WRKY transcription factor; SPDS, spermidine synthase; NHX, Na^+^/H^+^ exchanger.

Future research should prioritize three areas. First, well-controlled dose–response experiments should directly compare Put, Spd, and Spm within the same genotype, salinity regime, developmental stage, and application framework to distinguish compound-specific effects from experimental-context effects; where feasible, these designs should incorporate targeted manipulation of polyamine metabolism, transport, or signaling to strengthen causal interpretation. Second, longer-term and field-relevant experiments should determine whether the physiological benefits observed under controlled conditions persist through reproductive development and translate into yield-level responses. Third, primary studies should adopt standardized reporting of treatment structure, means, SD or SE, sample sizes, experimental replication, and preferably machine-readable data to improve reproducibility and enable more reliable quantitative synthesis. The resulting evidence hierarchy is summarized in Figure 6.

Taken together, the evidence supports the hierarchical framework summarized in Figure 6, in which the most reproducible effects occur at the level of oxidative damage and selected homeostatic responses. In contrast, downstream photosynthetic and growth responses are increasingly context-dependent. The principal biological modifiers of this hierarchy are discussed in Section 4.1, Section 4.2 and Section 4.3.

### 4.5. Limitations

The integrated interpretation has several important limitations. Independent family numbers remain small for ion balance, RWC, chlorophyll, A/Pn, and biomass; Spm is underrepresented relative to Spd and Put; several eligible studies reported means without defensible variance estimates; and many experiments used seedlings, short exposure periods, or controlled-environment conditions. Consequently, the conservative family-level synthesis reduces pseudoreplication but cannot compensate for limited independent evidence or incomplete statistical reporting. Importantly, responses observed under controlled laboratory or greenhouse conditions cannot be assumed to translate directly to field-grown plants, where environmental variability, soil heterogeneity, longer stress exposure, and reproductive development may modify treatment effects. Field-based and yield-level validation is therefore required before these findings can be generalized to agronomic conditions.

A single reviewer conducted the initial study selection and primary data extraction, which introduces a potential risk of selection and extraction error. To strengthen quality control, a second reviewer retrospectively and independently verified a random subset of the retained studies, and we also performed structured computational consistency checks. However, this retrospective verification did not constitute prospective duplicate screening or complete independent duplicate extraction; therefore, residual reviewer-related error or bias cannot be completely excluded. The review was not prospectively registered, and no formal study-level risk-of-bias instrument was applied. This decision reflected the substantial heterogeneity in experimental designs, reporting structures, and physiological endpoints across the retained literature, for which no single standard instrument could be applied consistently without substantial subjective adaptation. Nevertheless, the absence of a formal risk-of-bias assessment limits inference regarding the internal validity of individual experiments, and the retrospective auditing and computational quality-control procedures used here should not be interpreted as substitutes for formal risk-of-bias assessment. The literature search was also restricted to Scopus and Web of Science, so relevant studies indexed exclusively elsewhere may have been missed.

Taken together, the evidence indicates that exogenous polyamines most consistently stabilize redox status and ion–water homeostasis under salinity. In contrast, improvements in photosynthesis and growth are more conditional and depend on experimental context. The principal practical research gap is therefore not whether polyamines can mitigate components of salt stress, but which combinations of polyamine identity, dose, application strategy, stress intensity, genotype, developmental stage, and exposure duration translate these upstream benefits into sustained growth and yield. Addressing this gap will require longer-term, field-based experiments with reproductive and yield-level endpoints.

## 5. Conclusions

Across the current evidence base, four core inferences emerge. First, exogenous polyamines most consistently modulate redox status and ion–water homeostasis under salinity. Second, these upstream protective effects do not necessarily translate into sustained photosynthetic or growth recovery, which remains strongly dependent on experimental and biological context. Third, current evidence does not support a generally superior effect of Put, Spd, or Spm, indicating that polyamine identity must be interpreted together with dose, stress intensity and duration, genotype, tissue, developmental stage, and application strategy. Fourth, the effectiveness of exogenous polyamines is therefore better understood as a conditional physiological response than as a universal mechanism of salinity tolerance. Future research should prioritize well-controlled dose–response experiments directly comparing Put, Spd, and Spm within the same experimental framework, longer-term and field-relevant studies incorporating reproductive and yield-level outcomes, and standardized reporting of experimental design and statistical data. Targeted manipulation of polyamine transport, metabolism, and signaling should also be incorporated into these experimental frameworks where feasible to distinguish causal regulatory roles from responses associated with polyamine accumulation. Overall, current evidence supports exogenous polyamines primarily as modulators of redox and ion–water homeostasis under salinity, whereas translating these effects into sustained photosynthetic and growth benefits remains strongly context-dependent.

## Figures and Tables

**Table 1:** Quantitative synthesis by outcome. MDA and H_2_O_2_ are principal publication-family-aware multilevel estimates; O_2_•^−^ and the antioxidant enzymes use the same multilevel structure. Physiological outcomes supported by only four or five independent families use one frozen family mean per experimental/publication family and Student’s *t* intervals. Heterogeneity components and full reproducibility results are reported in the Results, Data S1 (Tables S2, S3 and S9), and the supplied analysis code. Estimates based on only four or five independent families should be regarded as preliminary.

Outcome	k Families	Contrasts	Metric	Estimate	95% CI/Sensitivity	Interpretation
MDA	15	61	Protective lnRR	0.214	0.138–0.289	19.2% reduction; robust
H_2_O_2_	12	41	Protective lnRR	0.296	0.168–0.424	25.6% reduction; robust
O_2_•^−^	5	18	Protective lnRR	0.181	−0.006–0.369	Protective mean; CI includes zero
SOD	6	21	Raw lnRR	0.145	−0.015–0.305	Context-dependent
CAT	7	23	Raw lnRR	0.120	−0.013–0.253	Context-dependent
APX	7	18	Raw lnRR	0.110	−0.030–0.250	Context-dependent
POD/POX	5	18	Raw lnRR	0.079	−0.067–0.224	Context-dependent
Ion balance	5	29	Harmonized lnRR	0.549	0.201–0.896	Favorable but variable; preliminary (k = 5)
RWC	4	15	lnRR	0.123	0.005–0.241	Modest favorable response; preliminary (k = 4)
Total chlorophyll	4	13	lnRR	0.230	−0.025–0.485	Suggestive; CI includes null; preliminary (k = 4)
A/Pn	4	14	lnRR	0.144	−0.507–0.795	No consistent average rescue; preliminary (k = 4)
Dry biomass	4	11	lnRR	0.382	0.171–0.594	Favorable; preliminary (k = 4)

Abbreviations: APX, ascorbate peroxidase; A/Pn, net CO_2_ assimilation/net photosynthetic rate; CAT, catalase; CI, confidence interval; H_2_O_2_, hydrogen peroxide; lnRR, natural logarithm of the response ratio; MDA, malondialdehyde; O_2_•^−^, superoxide anion; POD/POX, peroxidase; RWC, relative water content; SOD, superoxide dismutase.

## Data Availability

All datasets associated with this systematic review, evidence map, and family-aware quantitative synthesis are publicly accessible on Zenodo (DOI: 10.5281/zenodo.22289249). The repository contains Supplementary_Data_S1_evidence_synthesis_v9_REVISED.xlsx, which details the search workflow and provides metadata for the studies used in the family-aware quantitative synthesis. Additionally, Supplementary_Data_S2_contrast_matrix_444_REVISED.xlsx supplies study-level metadata for the quantitative synthesis, covering stress categories, response groups, effect-measure structures, benefit-direction rules, and aggregation methods. The Python analysis code used to reproduce the quantitative analyses is available from the corresponding author upon reasonable request. This study did not generate any novel experimental biological data.

## Supplementary Materials

The following supporting information can be downloaded at: https://www.mdpi.com/article/10.3390/antiox15091165/s1. Data S1: Evidence-synthesis workbook, including study screening, evidence classification, eligibility criteria, model summaries, reference audit, and reproducibility information [1,2,3,4,5,6,7,8,9,10,11,12,13,14,15,16,17,18,19,20,21,22,23,24,25,26,27,28,29,30,31,32,33,34,35,36,37,38,39,40,41,42,43,44,45,46,47,48,49,50]; Data S2: Complete quantitative contrast matrix and family-level inputs for the retained studies [2,3,6,7,18,19,20,21,22,23,24,25,26,27,28,29,30,31,32,33,34,35,36,37,38,39,40,41,42,43,44,45,46,47]; File S1: PRISMA 2020 checklist [17].

## References

[1] Shabala S., Cuin T.A., Pottosin I. (2007). Polyamines prevent NaCl-induced K^+^ efflux from pea mesophyll by blocking non-selective cation channels. FEBS Lett..

[2] Zhao X., Zhang Y., Zhang X., Shan C. (2023). Putrescine improves salt tolerance of wheat seedlings by regulating ascorbate and glutathione metabolism, photosynthetic performance, and ion homeostasis. Plant Soil Environ..

[3] Xue T.Y., Wan H.P., Wu X.M., Liu L., Chen J.D., Yu Y., Dai X.G., Dong Y.H., Zeng C.L. (2025). Exogenous spermidine (Spd) alleviates NaCl-induced injury effects by improving photosynthesis and oxidative stress tolerance in *Brassica napus* seedlings. Not. Bot. Horti Agrobo..

[4] Yamaguchi K., Takahashi Y., Berberich T., Imai A., Miyazaki A., Takahashi T., Michael A.J., Kusano T. (2006). The polyamine spermine protects against high salt stress in *Arabidopsis thaliana*. FEBS Lett..

[5] Hu X.H., Zhang Y., Shi Y., Zhang Z., Zou Z.R., Zhang H., Zhao J.Z. (2012). Effect of exogenous spermidine on polyamine content and metabolism in tomato exposed to salinity-alkalinity mixed stress. Plant Physiol. Biochem..

[6] Nahar K., Hasanuzzaman M., Rahman A., Alam M.M., Mahmud J.-A., Suzuki T., Fujita M. (2016). Polyamines confer salt tolerance in mung bean (*Vigna radiata* L.) by reducing sodium uptake, improving nutrient homeostasis, antioxidant defense, and methylglyoxal detoxification systems. Front. Plant Sci..

[7] Hai X., Mi J., Zhao B., Zhang B., Zhao Z., Liu J. (2022). Foliar application of spermidine reduced the negative effects of salt stress on oat seedlings. Front. Plant Sci..

[8] Maiale S., Sánchez D.H., Guirado A., Vidal A., Ruiz O.A. (2004). Spermine accumulation under salt stress. J. Plant Physiol..

[9] Pandolfi C., Pottosin I., Cuin T., Mancuso S., Shabala S. (2010). Specificity of polyamine effects on NaCl-induced ion flux kinetics and salt stress amelioration in plants. Plant Cell Physiol..

[10] Moschou P.N., Paschalidis K.A., Delis I.D., Andriopoulou A.H., Lagiotis G.D., Yakoumakis D.I., Roubelakis-Angelakis K.A. (2008). Spermidine exodus and oxidation in the apoplast induced by abiotic stress is responsible for H_2_O_2_ signatures that direct tolerance responses in tobacco. Plant Cell.

[11] Yang J., Wang P., Li S., Liu T., Hu X. (2022). Polyamine oxidase triggers H_2_O_2_-mediated spermidine improved oxidative stress tolerance of tomato seedlings subjected to saline-alkaline stress. Int. J. Mol. Sci..

[12] Tun N.N., Santa-Catarina C., Begum T., Silveira V., Handro W., Floh E.I.S., Scherer G.F.E. (2006). Polyamines induce rapid biosynthesis of nitric oxide (NO) in *Arabidopsis thaliana* seedlings. Plant Cell Physiol..

[13] Tanou G., Ziogas V., Belghazi M., Christou A., Filippou P., Job D., Fotopoulos V., Molassiotis A. (2014). Polyamines reprogram oxidative and nitrosative status and the proteome of citrus plants exposed to salinity stress. Plant Cell Environ..

[14] Shang C., Liu X., Chen G., Li G., Hu S., Zheng H., Ge L., Long Y., Wang Q., Hu X. (2024). SlWRKY81 regulates Spd synthesis and Na^+^/K^+^ homeostasis through interaction with SlJAZ1 mediated JA pathway to improve tomato saline-alkali resistance. Plant J..

[15] Alet A.I., Sánchez D.H., Cuevas J.C., Marina M., Carrasco P., Altabella T., Tiburcio A.F., Ruiz O.A. (2012). New insights into the role of spermine in *Arabidopsis thaliana* under long-term salt stress. Plant Sci..

[16] Recalde L., Vázquez A., Groppa M.D., Benavides M.P. (2018). Reactive oxygen species and nitric oxide are involved in polyamine-induced growth inhibition in wheat plants. Protoplasma.

[17] Page M.J., McKenzie J.E., Bossuyt P.M., Boutron I., Hoffmann T.C., Mulrow C.D., Shamseer L., Tetzlaff J.M., Akl E.A., Brennan S.E. (2021). The PRISMA 2020 statement: An updated guideline for reporting systematic reviews. BMJ.

[18] Li J., Hu L., Zhang L., Pan X., Hu X. (2015). Exogenous spermidine is enhancing tomato tolerance to salinity–alkalinity stress by regulating chloroplast antioxidant system and chlorophyll metabolism. BMC Plant Biol..

[19] Zeid I.M. (2004). Response of bean (*Phaseolus vulgaris*) to exogenous putrescine treatment under salinity stress. Pak. J. Biol. Sci..

[20] Duan J., Li J., Guo S., Kang Y. (2008). Exogenous spermidine affects polyamine metabolism in salinity-stressed *Cucumis sativus* roots and enhances short-term salinity tolerance. J. Plant Physiol..

[21] Zhang Y., Zhang L., Hu X.H. (2014). Exogenous spermidine-induced changes at physiological and biochemical parameters levels in tomato seedling grown in saline-alkaline condition. Bot. Stud..

[22] Jain V., Vart S., Verma E., Malhotra S.P. (2015). Spermine reduces salinity-induced oxidative damage by enhancing antioxidative system and decreasing lipid peroxidation in rice seedlings. J. Plant Biochem. Biotechnol..

[23] Shu S., Yuan Y.H., Chen J., Sun J., Zhang W.H., Tang Y.Y., Zhong M., Guo S. (2015). The role of putrescine in the regulation of proteins and fatty acids of thylakoid membranes under salt stress. Sci. Rep..

[24] Li S., Jin H., Zhang Q. (2016). The effect of exogenous spermidine concentration on polyamine metabolism and salt tolerance in zoysiagrass (*Zoysia japonica* Steud) subjected to short-term salinity stress. Front. Plant Sci..

[25] Rohman M.M., Islam T., Mohi-Ud-Din M., Islam M.R., Molla M.R., Hossain M.G., Chowdhury M.A.Z. (2017). Use of spermidine reduced the oxidative damage in onion seedlings under salinity by modulating antioxidants. Afr. J. Agric. Res..

[26] Siddiqui M.H., Alamri S.A., Al-Khaishany M.Y., Al-Qutami M.A., Ali H.M., Al-Rabiah H., Kalaji H.M. (2017). Exogenous application of nitric oxide and spermidine reduces the negative effects of salt stress on tomato. Hortic. Environ. Biotechnol..

[27] ElSayed A.I., Rafudeen M.S., El-Hamahmy M.A.M., Odero D.C., Hossain M.S. (2018). Enhancing antioxidant systems by exogenous spermine and spermidine in wheat (*Triticum aestivum*) seedlings exposed to salt stress. Funct. Plant Biol..

[28] El Sayed A.I., El-Hamahmy M.A.M., Rafudeen M.S., Ebrahim M.K.H. (2019). Exogenous spermidine enhances expression of Calvin cycle genes and photosynthetic efficiency in sweet sorghum seedlings under salt stress. Biol. Plant..

[29] Islam M.A., Pang J.-H., Meng F.-W., Li Y.-W., Xu N., Yang C., Liu J. (2020). Putrescine, spermidine, and spermine play distinct roles in rice salt tolerance. J. Integr. Agric..

[30] Wu Z., Wang J., Yan D., Yuan H., Wang Y., He Y., Wang X., Li Z., Mei J., Hu M. (2020). Exogenous spermidine improves salt tolerance of pecan-grafted seedlings via activating antioxidant system and inhibiting the enhancement of Na^+^/K^+^ ratio. Acta Physiol. Plant..

[31] Chen T., White J.F., Li C., Nan Z. (2021). Exogenous spermidine enhances *Epichloë* endophyte-induced tolerance to NaCl stress in wild barley (*Hordeum brevisubulatum*). Plant Soil.

[32] Farsaraei S., Mehdizadeh L., Moghaddam M. (2021). Seed priming with putrescine alleviated salinity stress during germination and seedling growth of medicinal pumpkin. J. Soil Sci. Plant Nutr..

[33] Islam M.J., Ryu B.R., Azad M.O.K., Rahman M.H., Rana M.S., Lim J.-D., Lim Y.-S. (2021). Exogenous putrescine enhances salt tolerance and ginsenosides content in Korean ginseng (*Panax ginseng* Meyer) sprouts. Plants.

[34] da Silva T.I., Dias M.G., de Araújo N.O., de Sousa Santos M.N., Cruz R.R.P., Dias T.J., Ribeiro W.S., Grossi J.A.S., Barbosa J.G. (2022). Spermine reduces the harmful effects of salt stress in *Tropaeolum majus*. Physiol. Mol. Biol. Plants.

[35] Korbas A., Kubiś J., Rybus-Zając M., Chadzinikolau T. (2022). Spermidine modify antioxidant activity in cucumber exposed to salinity stress. Agronomy.

[36] El-Beltagi H.S., El-Yazied A.A., El-Gawad H.G.A., Kandeel M., Shalaby T.A., Mansour A.T., Al-Harbi N.A., Al-Qahtani S.M., Alkhateeb A.A., Ibrahim M.F.M. (2023). Synergistic impact of melatonin and putrescine interaction in mitigating salinity stress in snap bean seedlings: Reduction of oxidative damage and inhibition of polyamine catabolism. Horticulturae.

[37] Pascual L.S., López-Climent M.F., Segarra-Medina C., Gómez-Cadenas A., Zandalinas S.I. (2023). Exogenous spermine alleviates the negative effects of combined salinity and paraquat in tomato plants by decreasing stress-induced oxidative damage. Front. Plant Sci..

[38] Uzma J., Talla S.K., Mamidala P. (2023). Insights into the impact of spermidine in reducing salinity stress in *Gerbera jamesonii*. J. Appl. Biol. Biotechnol..

[39] Bahrasemani S., Seyedi A., Fathi S., Jowkar M. (2024). The seed priming using putrescine improves germination indices and seedlings morphobiochemical responses of indigo (*Indigofera tinctoria*) under salinity stress. J. Med. Plants By-Prod..

[40] Bazvand F., Eisvand H.R., Daneshvar M., Rahimi-Moghaddam S., Paravar A. (2024). Can exogenous application of putrescine and priming modulate salinity stress in *Camelina sativa* L.?. Ind. Crops Prod..

[41] Mohammadi M., Nezamdoost D., Khosravi Far F., Zulfiqar F., Eghlima G., Aghamir F. (2024). Exogenous putrescine application imparts salt stress-induced oxidative stress tolerance via regulating antioxidant activity, potassium uptake, and abscisic acid to gibberellin ratio in Zinnia flowers. BMC Plant Biol..

[42] Shen X., Dai S., Chen M., Huang Y. (2024). Spermidine augments salt stress resilience in rice roots potentially by enhancing OsbZIP73’s RNA binding capacity. BMC Plant Biol..

[43] Alharbi B.M., Ali M.A.A., Abdelaal K., Dessoky E.S., Al-Harbi N.A., AL-Balawi S.M., Anazi H.K., Darwish D.B.E., Al Kashgry N.A.T., Alayafi A.A.M. (2025). Spermidine (Spd) as a modulator of osmotic, redox and ion homeostasis in common bean seedlings under salinity stress: Physiological, biochemical and molecular aspects. Chil. J. Agric. Res..

[44] Hossain M.M., Kordrostami M., Goto F., Rahimi M. (2025). Spermine treatment improves salinity tolerance in *Plantago major* by altering growth parameters, biochemical profiles and gene expression. Sci. Rep..

[45] Hamidi Moghaddam A., Sorkhoeih M.K. (2025). Evaluation of putrescine’s effects on the growth, visual quality, and some physio-biochemical parameters of henna (*Lawsonia inermis* L.) under salinity stress conditions. BMC Plant Biol..

[46] Muradoğlu F., Batur Ş., Hasanov M., Güler E. (2025). Putrescine eases saline stress by regulating biochemicals, antioxidative enzymes, and osmolyte balance in hydroponic strawberries (cv. Albion). Physiol. Plant..

[47] Yang X.F., Yao C.J., Li Y.J., Zhang S.J. (2025). Effect of exogenous putrescine on wheat seed germination and physiological characteristics under salt stress. Appl. Ecol. Environ. Res..

[48] Xu J., Kang Z., Zhu K., Zhao D., Yuan Y., Yang S., Zhen W., Hu X. (2021). RBOH1-dependent H_2_O_2_ mediates spermine-induced antioxidant enzyme system to enhance tomato seedling tolerance to salinity–alkalinity stress. Plant Physiol. Biochem..

[49] Liu C., Atanasov K.E., Tiburcio A.F., Alcázar R. (2019). The polyamine putrescine contributes to H_2_O_2_ and RbohD/F-dependent positive feedback loop in *Arabidopsis* PAMP-triggered immunity. Front. Plant Sci..

[50] Santos Filho F.B., da Silva T.I., Dias M.G., Grossi J.A.S. (2022). Polyamines mitigate the harmful effects of salt stress on the growth and gas exchange of nasturtium. Ciênc. Agrotecnol..

